# Evaluation of Social Values for Ecosystem Services in Urban Riverfront Space Based on the SolVES Model: A Case Study of the Fenghe River, Xi’an, China

**DOI:** 10.3390/ijerph18052765

**Published:** 2021-03-09

**Authors:** Zhicheng Zhang, Hongjuan Zhang, Juan Feng, Yirong Wang, Kang Liu

**Affiliations:** 1 College of Urban and Environmental Sciences, Northwest University, Xi’an 710127, China; zhangzhicheng1@stumail.nwu.edu.cn (Z.Z.); 201820796@stumail.nwu.edu.cn (J.F.); 201831849@stumail.nwu.edu.cn (Y.W.); 2 Key Research Institute of Yellow River Civilization and Sustainable Development & Collaborative Innovation Center on Yellow River Civilization Jointly Built by Henan Province and Ministry of Education, Henan University, Kaifeng 475001, China; 10340044@vip.henu.edu.cn; 3 National Forestry and Grassland Administration Urban Forest Ecosystem Research Station, Xi’an 710127, China

**Keywords:** SolVES model, landscape space, participatory mapping, environmental variables

## Abstract

Urban riverfront space has diversified ecosystem services, but due to excessive changes in the geographical environment, such as drastic changes in land use, people gain social value at a great ecological cost. Obtaining benefits from the ecosystem in this way is not sustainable. Therefore, this paper uses the SolVES model to evaluate the social value of ecosystem services on the east bank of the Fenghe River, while also studying the contribution of different environmental variables to social value. The main results are as follows. (1) Environmental variables affect the spatial distribution characteristics of social value. The distance to water (DTW) means the social value was distributed in strips, and the distance to road (DTR) concentrated the social value along the road. The landscape type (LT) means the social value was concentrated in the landscape space. (2) When DTW, DTR, and LT were collectively used as environmental variables, the distribution characteristics of various social values were similar to when LT was used as the only environmental variable. (3) The results of MaxEnt show that LT made a greater contribution to the aesthetic, recreation, therapeutic, and historic values, and was the largest contribution factor to the aesthetic, therapeutic, and historic values, with contribution rates of 47.6, 50.5, and 80.0%, respectively. DTW is the factor that contributed the most to recreation, with a contribution rate of 43.1%. Improving social value based on the influence and contribution of environmental variables can reduce the damage to the ecological environment caused by changes in geographic factors. This is sustainable for both the ecosystem and the services it provides to mankind.

## 1. Introduction

Since the SCEP (Study of Critical Environmental Problems) first clearly proposed ecosystem services (ES) in 1970 [1,2], scholars have been discussing the concept [3,4,5]. It is now recognized by most scholars that ES are products or benefits that humans obtain directly or indirectly through the ecosystem to maintain human life [6]. They not only provide humans with all kinds of material resources needed for production and life, but also meet humans’ spiritual, cultural, and emotional needs; ES can also adjust the local climate to make it suitable for human survival [7]. ES are proposed from a human perspective so that the services and products provided can be effectively used by humans. Therefore, ES must be part of the ecological function used by humans [8,9,10]. In 2003, the Millennium Ecosystem Assessment (MEA) established a comprehensive ecosystem assessment framework and divided ES into four categories: “provisioning services,” “regulating services,” “supporting services,” and “cultural services” [7,11]. This framework shows that a comprehensive assessment of ecosystems requires extensive natural and social information [12]. Since this period, when researching ES, researchers have focused more on the relationship between the functional value of ES and the economy or society. The assessment of ecosystems and their services is used to guide or solve some decision-making and planning issues. As Maes stated, “Assessment is the analysis and review of information to help those responsible for assessing possible actions or thinking about problems” [13,14]. Ecosystem assessment can be seen as a synthesis of ecosystem data based on policy issues [14]. Based on this, many countries have issued national ecosystem assessments, and established ecological databases as an important knowledge base for maintaining biodiversity and ecosystem services [15]. This also provides guidance and a data guarantee for decision-making, ecological planning, and green infrastructure construction [16].

In the 21st century, although good results have been achieved in the evaluation of ES, due to the inherent complexity of the ecosystem, there are still many key issues that need to be resolved in the current research on ES value evaluation. For example, the social value of recreation in the market economy cannot be reflected. Therefore, the evaluation of the overall value of ES cannot be completely dependent on the economic value evaluation method, and a nonmonetary form of evaluation is needed to evaluate the value of the social attributes of ES. The emergence of the SolVES model (Social Values for Ecosystem Services model, verified by Sherrouse and Semmens [17]) is the transformation of ES evaluation from a single economic value evaluation to a nonmonetized spatial distribution evaluation. This is a major breakthrough for researchers in terms of how to comprehensively and reasonably evaluate the value of ES [18,19]. In 2011, Sherrouse et al. [20] quantified the social value of ES in the Santa Isabel national forest in Colorado, USA, which set off a wave of social value assessment. For example, Riper et al. evaluated the social value for ES of Hinchinbrook Island national forest in Australia in 2012 [21]. In addition, the application of the value transfer method extends the original data to a larger area [22], and this method has been widely used in the economic evaluation of ES [23,24,25]. The SolVES model also has a social value transfer function, and some scholars also use this method to evaluate the social value for ES [18,26].

Although many scholars have evaluated the social values for certain types of areas such as forest parks [20,21,26], wetland parks [27], and economic regions [28], there are still a few evaluations of the social value of urban riverfront spaces. Urban riverfront spaces often exhibit a unique strip form, which causes problems such as simple landscape types and single service functions [29]. Therefore, it is of practical significance to evaluate the social value of ES in urban riverfront spaces in terms of the rational configuration of landscape types and the diversification of service functions. In addition, many scholars tend to focus on the relationship between social value and environmental variables when evaluating social value [29,30]. A few scholars described the changes in the distribution of social value and the value index when a certain environmental variable is missing [28]. For example, Cheng et al. [29] used distance to water (DTW) and distance to road (DTR) as environmental variables to evaluate the social value of the Huangpu River waterfront space. Zhao et al. [28] found that, during the operation of the SolVES model, land use and land cover (LULC) have a greater impact on the results of the model when evaluating cultural services in the Guanzhong‒Tianshui economic region. When LULC is not used, the high and low values of the value index (VI) of the five cultural service indicators increase significantly and the distribution area expands. However, these results do not quantify the contribution of each environmental variable to social value, which may cause managers and planners to be unable to assign correct weights to various environmental factors in the region when considering plans. From a sustainability perspective, the rapid changes in environmental factors may bring about serious negative effects on the ecosystem [31] and destroy its integrity. Therefore, understanding how each environmental variable affects social value and their contribution to each type of social value can allow humans to reduce unnecessary changes to environmental factors and enjoy more services provided by the ecosystem. Considering that being close to water is a natural condition of riverfront space, and traffic directly or indirectly affects tourists’ satisfaction with social values [32], this paper selects DTW and DTR, which are closely related to urban riverfront space, as environmental variables. The importance of LULC in social value evaluation has been proven [28]. Therefore, this paper also uses the landscape type (LT), which is a special LULC based on the landscape function, as the environmental variable to evaluate the social values. In summary, this paper will compare the impact of each environmental variable on the distribution of social value and the value index and will further analyze the contribution of each variable to social value based on the quantitative results, under the premise that the three environmental variables work together.

This study has the following main purposes: (1) Assessing the social value of ES on the east bank of the Fenghe River, and then analyzing the impact of various environmental variables on the distribution of social values. (2) Studying the contribution of different environmental variables to various social values on the east bank of Fenghe River. In addition, based on the influence and contribution of different environmental variables to social values, we hope that we can find some promotion methods of the social values of urban riverfront space and a strategy of landscape optimization that is less costly to the ecosystem.

## 2. Materials and Methods

### 2.1. Study Area

**Figure 1:**
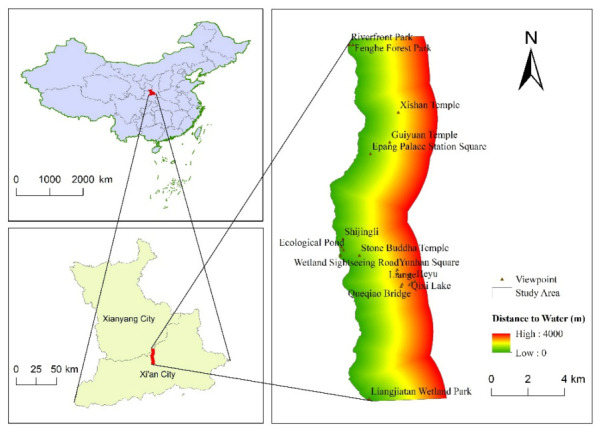
Location of the 4000-m buffer zone on the east bank of Fenghe River.

The Fenghe River originates from Fengyu, Chang’an District, Xi’an City, and merges into the Weihe River in Xianyang City, with a total length of 78 km [33]. The 4000-m buffer zone on the east bank of the Fenghe River is rich in landscape resources such as wetlands, forests, and relics (in this paper, relics refers to ancient buildings or ruins of historical significance). There are many scenic spots attracting tourists from all over the world. Therefore, the objective evaluation of the social value for ES of this area is extremely important for the rational use of its landscape resources. This paper comprehensively considers the location, as well as natural and social factors, and selects the Xi’an section of the Fenghe River as the research section based on the distance from the main urban area of Xi’an and tourists’ favorite scenic spots. Specifically, the upper reaches of the section of the Fenghe River are close to the Liangjiatan Wetland Park, and the lower reaches to the Fenghe Forest Park. The 4000-m buffer zone of the east bank of this section is the study area of this paper (Figure 1).

### 2.2. Survey Data Collection

The research data were collected through a field survey using the method of participatory mapping. This method allows respondents to allocate a fixed amount of money to different types of social values according to their personal wishes and allows them to mark the corresponding value points on the map [20]. In detail, we asked 10 investigators to randomly invite respondents to allocate 100 yuan to the seven social value types—aesthetic, economic, historic, recreation, spiritual, therapeutic, and future—based on their personal perception. Then, we asked these respondents to mark locations on the map that represent the social value types for which the allocated amount is greater than 0. These marked locations are social value points. When processing the data, we made the allocated results into a table and input it into ArcGIS (Esri, Redlands, CA, USA). We also input 5520 social value points into ArcGIS to obtain a layer of social value points by referring to the latitude and longitude on the map, landmark features, and the description or remarks of respondents. Integrating the method of participatory mapping into survey and data conversion is more meaningful than simple statistics, which only calculates the average value assigned by respondents to each social value. This is because the method can not only reflect the respondents’ perception of various social values but can also further integrate the SolVES model to expand the allocated results to the study area based on environmental variables to show the spatial distribution of these social values [26]. 

From 1 to 7 October 2020, we conducted the first stage of the field survey, which mainly used the form of field investigation to determine which scenic spots on the east bank of the Fenghe River are more popular with people. After completing the first phase of the survey, we started setting up the questionnaire. The questionnaire has three parts (Appendix A). The first part was a survey of respondents’ travel preferences and their satisfaction with travel on the east bank of the Fenghe River, such as the frequency of visits, travel companions, satisfaction with amusement facilities, etc. In the second part, respondents allocated 100 yuan to various social values based on their own perception and marked out social value points on the map. Taking into account that some respondents are not familiar with the map of the study area, we chose 20 well-known scenic spots on the east bank of the Fenghe River that were surveyed in the first stage and marked them on the map. The third part gathered the basic information of respondents, including gender, age, occupation, income, education, and residence. The second part of the questionnaire refers to the case of social value assessment for ES in the Santa Isabel National Forest Park in Colorado, USA [20]. From the social value types proposed [20], we selected seven types of social value related to the characteristics of the study area for respondents to choose from. The description of social value has been improved to make it better understood by Chinese people (Table 1). From 15 to 20 October 2020, we conducted the second stage of the survey. We sent out 60 questionnaires and asked participants to suggest improvements to the questionnaire. More than 90% of the respondents thought that the questions in the questionnaire were easy to understand.

Starting on November 1, 2020, we conducted a 30-day random survey. We set up one or two investigators at the exit, visitor center, and parking lot of each scenic spot. People who have finished sightseeing eventually return to these places to take a bus or private car. At the same time, these people can fully express their experience [26]. After explaining our identity and purpose, we introduced and explained the contents of the questionnaire to respondents. We invited about 750 people to fill out the questionnaire, 540 of whom agreed. Of these, 508 responses were valid. Therefore, the response rate of this survey was 72.00%, and the effective rate was 94.07%.

### 2.3. Spatial Data

**Figure 2:**
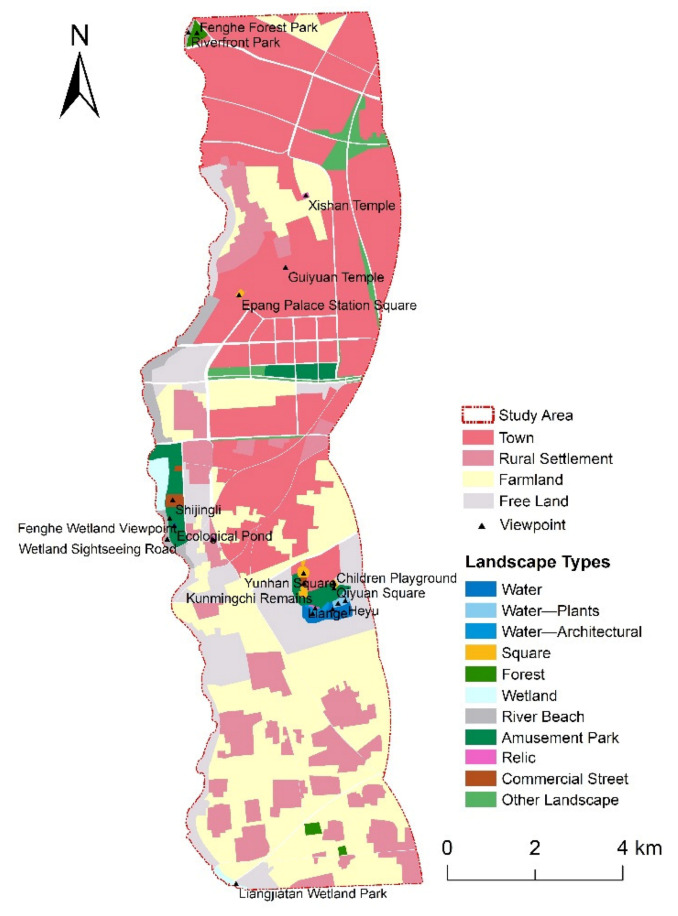
Classification of landscape types.

The spatial data required in this paper include the study area boundary, the social value points, and the environmental variables. The sources of these data are shown in Table 2. The boundary of the 4000-m buffer zone of the east bank of the Fenghe River was used as the study area boundary layer, and the social value points obtained through the second part of the questionnaire were digitally processed in ArcGIS 10.2 to obtain the social value point layer. At the same time, we selected the distance to water (DTW), distance to road (DTR), and landscape types (LT), which are closely related to the urban riverfront space, as the geographical environment layers of this study. DTW and DTR are calculated by the Euclidean distance tool in ArcGIS 10.2, and ArcGIS rasterizes these calculation results. The rasterized results of DTW and DTR are used as the geographical environment layer in this paper. Combined with the existing landscape resources of the study area, the landscape types in the General Planning Standards for Scenic Spots in China (GBT 50298-2018) are screened and adjusted appropriately. Eleven types of landscapes were selected, including water, water‒plants (W‒P), water‒architectural (W‒A), forest, wetland, river/beach, amusement park (A‒P), square, commercial street (C‒S), relic, and other landscape (O‒L) (Figure 2). We used ArcGIS 10.2 to visually interpret the satellite images in the study area to obtain landscape type vector data, converted the vector data into a raster, and finally got the LT raster results. The raster data of LT are also used as the geographical environment layer in this paper.

MaxEnt embedded in SolVES generates models for the social value types selected by the user. Along with these models, MaxEnt produces additional statistics describing the performance of each model. Included in these is the AUC statistic (the area under the receiver operating characteristic (ROC) curve) [34]. ROC is a curve in which the false positive rate (specificity or commission error) of the predicted class membership is plotted on the x-axis and the true positive rate (sensitivity or omission error) is plotted on the y-axis. MaxEnt does not rely on true absence points, so the ROC curves it generates plot the fractional predicted area on the x-axis, which considers the fact that Maxent is analyzing presence random data instead of presence absence data [35,36]. According to the verification of Sherrouse and Semmens [17], the value of the test AUC can be used to measure the accuracy of the SolVES model’s running results and the rationality of the environmental variable selection. The closer the test AUC is to 1, the better the evaluation effect [37]. When the value of the test AUC is 0.7 to 0.8, the evaluation result of social value is more accurate; when the value of the test AUC is 0.8 to 0.9, the evaluation result is very accurate; when the value of the test AUC is greater than 0.9, the evaluation result is extremely accurate [38,39]. The test AUC produced by each variable is shown in Table 3. All test AUC values are greater than 0.9. However, if the environmental variables are highly correlated, the above assessment method may not be correct [35]. Therefore, before analysis, we conducted a collinearity test on DTW, DTR, and LT. The test results (Appendix B) show that there is no multicollinearity in the three environmental variables, which meets the modeling standards. The value of the test AUC and the results of the collinearity analysis show that the DTW‒DTR‒LT model (DTW, DTR, and LT are used together as environmental variables), DTW model (DTW is used as the only environmental variable), DTR model (DTR is used as the only environmental variable), and LT model (LT is used as the only environmental variable) all have good model running results. Therefore, we have chosen these four models to facilitate the study of the social value for ES based on different environmental variables.

### 2.4. SolVES as a Value Assessment Tool

The SolVES model developed by the United States Geological Survey and Colorado State University can be used to evaluate and quantify the social value of ES such as aesthetics, biodiversity, and recreation [17,40]. The SolVES model uses the average nearest neighbor tool in ArcGIS to perform average nearest neighbor analysis on selected social value points of the seven social value types, and it expresses the spatial clustering results of each social value type through R value and Z score. It is generally believed that R < 1 indicates that the distribution of corresponding social value types has spatial agglomeration [29]. In addition, based on a survey of respondents’ preferences, the SolVES model uses a kernel density analysis tool to perform a weighted kernel density analysis on the social value points marked by the respondent to obtain a kernel density surface. In this process, the total amount allocated by the respondent to each social value type is the weight. Then, SolVES identifies the maximum weighted kernel density value in each social value surface to obtain the overall maximum grid value. Finally, the nuclear density surface is divided by the maximum grid value surface to obtain a nuclear density value index layer standardized to 0–10. These normalized surfaces are standardized to an exponential scale with 10-point values to generate a constant grid of the maximum value in each value index grid [34]. The exponential scale with 10-point values is the value index (VI), and the highest VI of each social value type is the maximum value index (M-VI). The results of M-VI indicate the degree of importance of various social value types. The greater the value, the higher the importance of the social value [40].

The model is composed of three submodels: the social value model, value mapping model, and value transfer model. This paper uses a social value model and a value mapping model to evaluate the social value for ES. The social value model and the value mapping model need to be used in combination, and data such as environmental data, survey data, and study area boundaries are needed [41]. The model operation involves first using the social value model to select the stakeholder group and obtain the position of the maximum value and the determined highest rated value through a nuclear density analysis; secondly, the social values are selected by the value mapping model, and the VI is obtained by normalizing these values according to the maximum value. At the same time, the environmental change is calculated. Finally, the SolVES model uses the statistical function of MaxEnt (maximum entropy model) to enhance its expression [30]. MaxEnt randomly selects 25% of the distribution points from the environmental data imported by SolVES as the testing data, and the remaining 75% as the training data, and uses a jackknife test to determine the contribution of each environmental variable to the social value distribution. SolVES uses ArcGIS to output the calculation results of MaxEnt and obtains the relationship between VI and environmental variables [31].

### 2.5. Data Analysis

The data analysis can be divided as follows:

(1) We entered the collected valid questionnaires into SPSS and Excel, and counted the age, gender, travel season, travel satisfaction, etc. of the respondents to determine the demographic characteristics of the respondents.
(2) By using the SolVES model of ArcGIS 10.0, the average nearest neighbor tool was used to analyze the social value points labeled by each social value type, and the spatial clustering results of social value points on the east bank of the Fenghe River were obtained.
(3) By comparing the spatial distribution characteristics of various social values in the social value maps output by the SolVES model based on four environmental variables (DTW univariate; DTR univariate; LT univariate; and DTW, DTR, and LT integrated variables), the influences of these variables on the spatial distribution pattern of social value were determined.
(4) We imported the nonlinear data between mean VI and distance variables (DTW and DTR) output by SolVES into Origin software to obtain the fitted linear equations and the fitted curves. Then the correlation between the VI of various social values and the distance variables on the east bank of Fenghe River were analyzed by using these curves and equations. At the same time, we calculated the average VI of each landscape with the help of the zonal statistics tool in ArcGIS to analyze which type of landscape has a higher average VI.
(5) We used eight-neighbor to define the weight matrix and performed spatial autocorrelation statistics in GeoDa. Moran’s I, *p* values, and Z scores are used to analyze the spatial correlation between the distance variables (DTW and DTR) and VI when DTW, DTR, and LT were used together as environmental variables.
(6) We used the environmental variable contribution rate output from MaxEnt to determine the contribution rate of each variable to social value types when DTW, DTR, and LT were used together as environmental variables.

For spatial consistency, all raster layers were outputted for a cell size of 1000 m. The east bank of the Fenghe River maps had a scale of 1:1,000,000, and the recommended output resolution was 1000 m [35].

## 3. Results

### 3.1. Demographic Analysis of Respondents

Among the respondents, men accounted for 55.7% and women accounted for 44.3%; the ratio of men to women was 1.26:1. While 65.9% of the respondents were residents of Xi’an or Xianyang, 34.1% were from other cities; 48.8% of the respondents chose to travel with their families. Respondents with a high school degree or above accounted for 92.7%. The educational level of the respondents was generally higher, and the content of the questionnaire could be understood easily, which reduces the negative impact on the evaluation results caused by a misunderstanding of the questionnaire. The Likert-scale method (1–5 points) was used to evaluate respondents’ satisfaction with the east bank of the Fenghe River. The respondents’ overall experience of visiting the east bank of the Fenghe River scored 3.91. The overall satisfaction was relatively high. The satisfaction score for tourist facilities is 3.92, the satisfaction score for the tour environment is 3.95, and the satisfaction score for tourism attraction is 3.84. In addition, most respondents indicated that the traffic on the east bank of the Fenghe River is inconvenient, and the traffic facilities leading to the scenic spot should be improved.

### 3.2. Spatial Cluster Analysis

#### 3.2.1. Distribution of Social Value Points

**Figure 3:**
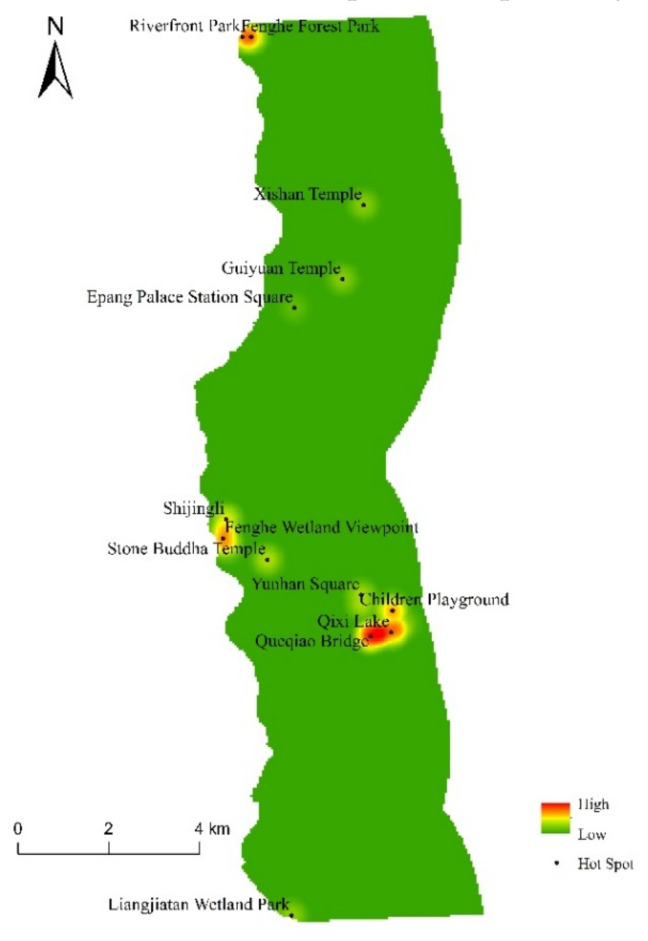
Spatial distribution density of social value points on the east bank of Fenghe River.

To a certain extent, the distribution of social value points can reflect the overall preference of respondents for scenic spots [34]. The SolVES model performs a nuclear density analysis on 5520 social value points to identify the hot spots on the east bank of the Fenghe River and obtain the spatial distribution of social value points. The results show that the hot spots are mainly concentrated in the Fenghe Forest Park and Fenghe Ecological Scenic Area, which are 0 to 1000 m away from the river, and the Kunmingchi Park, which is 3000 to 4000 m away from the river. Shijingli, Qixi Lake, and Queqiao Bridge are the most densely populated, with 397, 432, and 399 social value points, accounting for 7.19, 7.83, and 7.23% of the total number of social value points, respectively (Figure 3).

#### 3.2.2. Spatial Cluster Analysis of Social Value Based on Different Variables

From the average nearest neighbor analysis results (Table 4), it can be seen that the spatial distribution of the seven social value types on the east bank of the Fenghe River calculated by the four environmental models all belong to the spatial aggregation mode (R < 1). The M-VI of each social value types shows a certain degree of difference in different environmental models, and the order of the M-VI of the social value calculated by the four models also shows a certain degree of difference. The order of the M-VI of the seven social value types produced by the DTW model, DTR model, and DTW-DTR-LT model is aesthetic > recreation = therapeutic > historic > economic = future > spiritual. The order produced by the LT model is aesthetic > recreation > therapeutic = historic > economic = future > spiritual. However, the social value types with higher M-VI produced by the four models are the same: aesthetic, recreation, therapeutic, and historic (M-VI > 5). Therefore, this paper selects aesthetic, recreation, therapeutic, and historic for in-depth discussion.

### 3.3. Evaluation of the Social Value for ES

#### 3.3.1. Evaluation of Social Value Based on the DTW Model

**Figure 4:**
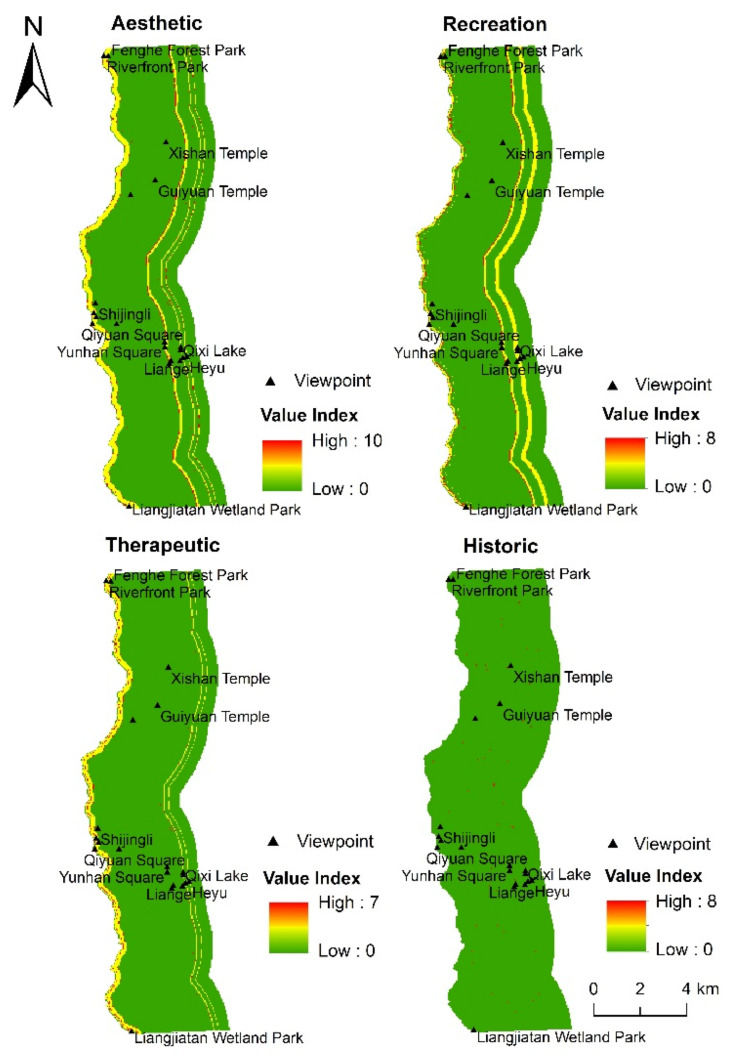
Distribution of social value based on distance to water.

**Figure 5:**
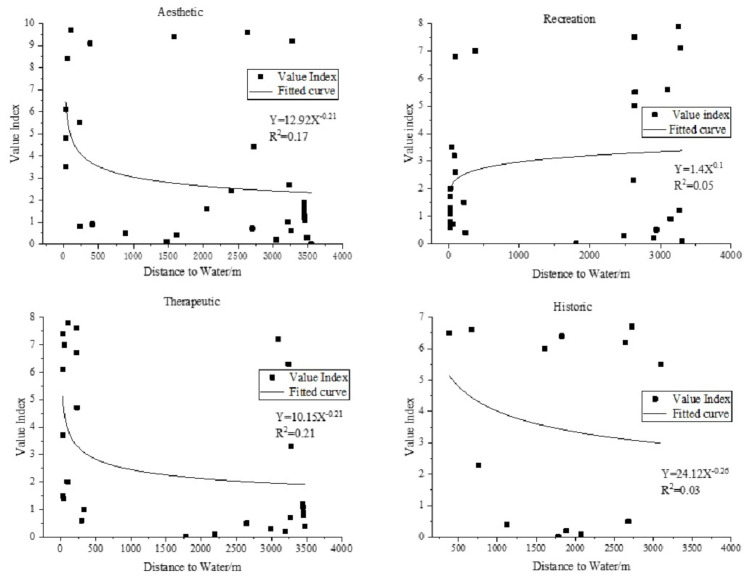
Statistics of the correlation between distance to water and value index.

We studied the relationship between the DTW and VI when the DTW is the only environmental variable (Figure 4 and Figure 5). It can be seen from Figure 4 that when the DTW is used as the only environmental variable, the VIs of the aesthetic, recreation, and therapeutic values show obvious striped distribution characteristics, and these striped spaces all pass through the denser areas of scenic spots on the east bank of the Fenghe River. The VI of historic does not show obvious distribution characteristics. It can be seen from Figure 5 that aesthetic, recreation, and therapeutic are mainly concentrated in the areas 0 to 500 m and 3000 to 3500 m away from the Fenghe River, while historic is relatively scattered in the space. In addition, it can be seen from the fitted curve of DTW and VI that on the east bank of the Fenghe River, the VIs of the aesthetic, therapeutic, and historic values are negatively correlated with DTW, and the VI of recreation is positively correlated with DTW.

#### 3.3.2. Evaluation of Social Value Based on the DTR Model

**Figure 6:**
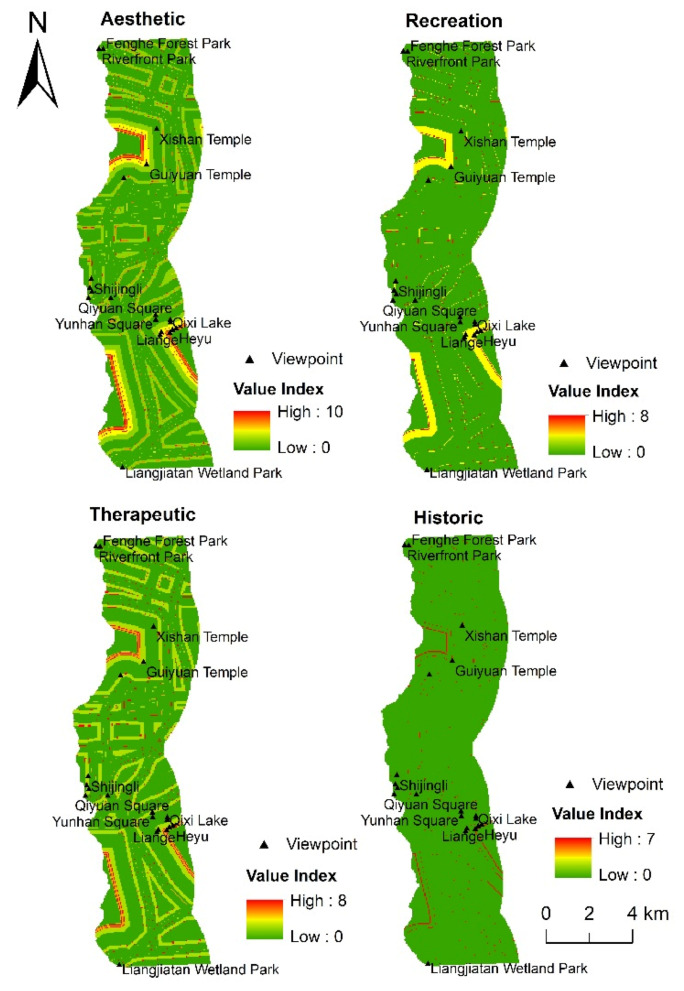
Distribution of social value based on distance to road.

**Figure 7:**
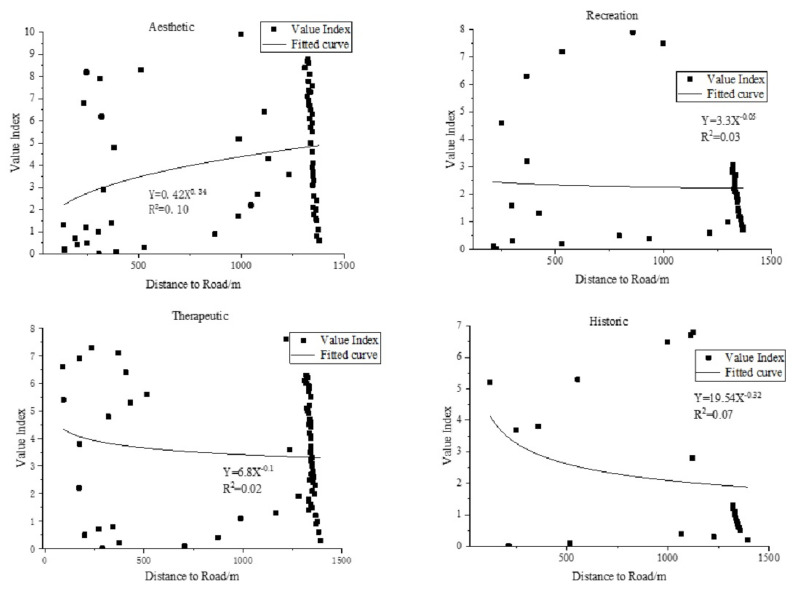
Statistics of the correlation between distance to road and value index.

We also studied the relationship between the DTR and VI when the DTR is the only environmental variable (Figure 6 and Figure 7). It can be seen from Figure 6 that the VIs of the aesthetic, recreation, and therapeutic values are mainly distributed along the roads in the study area, while the VI of the historic value does not show obvious distribution characteristics. At the same time, it can be seen from Figure 7 that aesthetic and therapeutic are mainly concentrated 0 to 500 m and 1250 to 1400 m from the road, while recreation is concentrated 1250 to 1400 m from the road. The fitted curve of the DTR and VI shows that the VIs of the recreation, therapeutic, and historic values are negatively correlated with the DTR, and the VI of the aesthetic value is positively correlated with the DTR.

#### 3.3.3. Evaluation of Social Value Based on the LT Model

**Figure 8:**
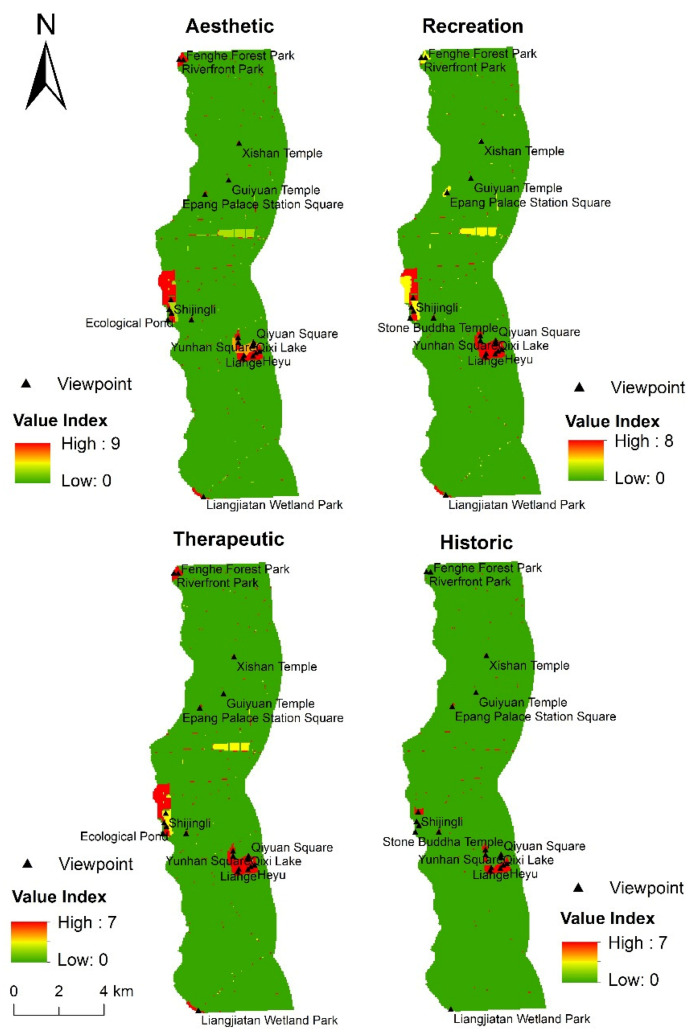
Distribution of social value based on landscape types.

When LT is used as the only environmental variable, the aesthetic, recreation, therapeutic, and historic values are mainly concentrated in areas with denser scenic spots (Figure 8). Among them, aesthetic and therapeutic are mainly concentrated in the Fenghe Ecological Scenic Area and Kunmingchi Park. Recreation and historic are mainly concentrated in Kunmingchi Park and Shijingli. The zonal statistics tool in ArcGIS 10.2 was used to count the mean VI, maximum VI, and minimum VI of the 11 landscape types. From the statistical results (Table 5), it can be seen that, for most landscape types, the maximum and mean values of the aesthetic value’s VI are higher than those of the other three social value types. Water, W‒P, and W‒A have the highest mean VIs of aesthetic, recreation, and therapeutic, and relic has the highest mean VI of aesthetic and historic. In addition, the mean VI of the aesthetic value in the spaces where square, wetland, relic, and C‒S are located is also higher, being 4.76, 3.23, 4.92, and 4.81, respectively.

#### 3.3.4. Evaluation of Social Value Based on the DTW-DTR-LT Model

**Figure 9:**
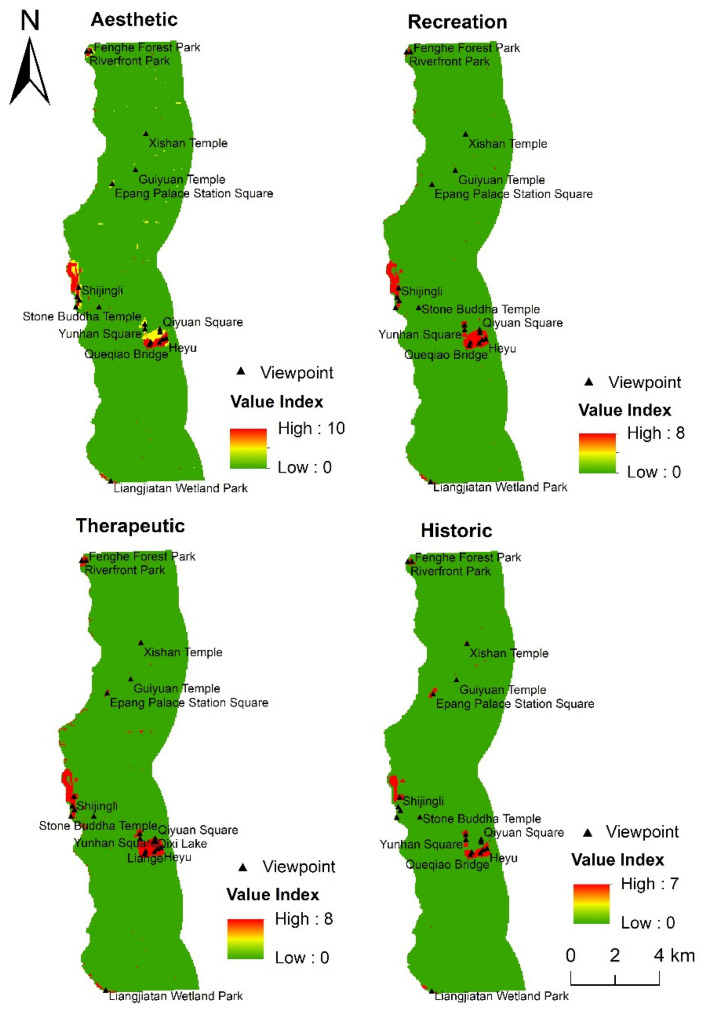
Distribution of social value based on distance to water, distance to road, and landscape types.

It can be seen from Figure 9 that, when DTW, DTR, and LT are collectively used as environmental variables, the distribution characteristics of the aesthetic, recreation, therapeutic, and historic values are similar to when LT is used as the only variable; that is, these social value types are mainly concentrated in areas with denser scenic spots. Among them, the aesthetic value is mainly concentrated in areas close to the river, such as Riverfront Park, Shijingli, Queqiao Bridge, and Qixi Lake. The recreation and therapeutic values are mainly concentrated in the Fenghe Ecological Scenic Area and Kunmingchi Park. The historic value is mainly concentrated in the Kunmingchi Park, Shijingli, and Epang Palace Station Square.

We used spatial autocorrelation statistics to analyze the spatial correlation between the distance variables (DTW and DTR) and VIs of the aesthetic, recreation, therapeutic, and historic values (Table 6). It is generally believed that 0 < |Moran’s I| < 1, and the closer |Moran’s I| is to 1, the more significant the spatial correlation; when Moran’s I > 0, there is a positive spatial correlation, and the spatial shape is an agglomerated distribution; when Moran’s I < 0, there is a negative spatial correlation, and the spatial shape is in a discrete pattern; when Moran’s I = 0, there is no spatial autocorrelation, and the spatial shape is a random pattern [30]. It can be judged by the *p* value and Z score (Table 6) that, except for historic VI and DTW, which are spatially randomly distributed, the confidence of the rest reaches 99%. Specifically, there is no spatial autocorrelation between the VI of historic and DTW; the spatial correlation between the VIs of the aesthetic, recreation, and therapeutic values and DTW is significantly negative; the spatial correlation between the VIs of four social value types and DTR is significantly positive.

SolVES used MaxEnt to analyze the contribution of each geographic environment factor to various social value types (Table 7). The contribution of LT to the aesthetic, therapeutic, and historic values is greater than that of DTW and DTR. Among them, the contribution of LT to historic is the largest, with a contribution rate of 80.0%, which is much greater than the contribution rate of DTW and DTR. DTW has the largest contribution to recreation, with a contribution rate of 43.1%. LT is second only to DTW, with a contribution rate of 39.1%, which is close to that of DTW. On the whole, LT contributes more than DTW or DTR to the four social value types, and DTR contributes the least.

## 4. Discussion

### 4.1. Responses of Social Values to Environmental Variables and Preferences of Respondents

The SolVES model expands the social value assigned by respondents to the entire study area based on environmental variables [26,35]. In other words, the evaluation results output by SolVES are mainly affected by the selected environmental variables, the location of social value points and the value assigned by respondents [30,34]. Respondents assign different monetary amounts to different social values, then express their spatial perception of these social values in the form of social value points, so that the VI of the social value corresponding to each point can be obtained [42]. However, in the process of assessment, different environmental variables were imported into the SolVES model, and the social value points and the value assigned results remained unchanged. This shows that the spatial distribution pattern of social value is only affected by environmental variables [35]. Therefore, it is feasible to use the method of importing single environmental variable into the SolVES model to study the influence of environmental variables on the spatial distribution pattern of social values. In terms of the results, when DTW, DTR, and LT are collectively used as environmental variables, the social value distribution pattern obtained by SolVES is closer to when LT is used as the only variable. This shows that, among the three environmental variables selected in this study, LT, as a special LULC, has the most significant impact on the spatial distribution pattern of social value. Especially for historic value, when DTW or DTR is used as the only environmental variable, it does not show a spatial distribution pattern consistent with the other three types of social values, but when LT is used as the only environmental variable or the three variables are collectively used, the spatial distribution pattern of the four types of social values are similar. Based on these results, it can be concluded that LT or LULC should be used as one of the environmental variables when evaluating the social value of urban riverfront space.

The preference of respondents is an important factor affecting the VI [42,43,44,45]. When we only use DTW, the VI in the area close to the river is generally higher, which is consistent with the findings of Sun et al. [42]. When we use LT for evaluation, the results of zonal statistics show that for water, water–plants, and water–architectural, the VI of social value except historic are higher. This shows that the respondents prefer the hydrophilic landscapes [43,46]. People’s preference for water can be explained by its ability to enhance the order and naturalness of the scene [47]. The water landscape gives people a sense of calm and stability, which is suitable for thinking or private conversations, and it has the functions of visual guidance and emotional extension [48]. A hydrophilic landscape has the function of separating land and water, and it has good visual factors for appreciation by water. The plant landscape coordinated with the width of the water surface can realize the ecological environment function of the hydrophilic landscape, and it forms a visual continuous space between water and land [49]. This also shows that the attractiveness of the landscape has a non-negligible influence on the preferences of people. In addition, the attractiveness of the landscape and environmental variables jointly affect the spatial distribution of the VI [42,45]. A good landscape has a strong attraction, and a reasonable space connection provides convenience for people to entered, and it is also easier for people to perceive social values [27,32]. This can explain why the VI of the area close to the road is higher, while the VI of the area far away from the road is almost zero. Many studies have the same results [29,34].

Certainly, it is not comprehensive to explain the evaluation results of the social value for ES only from the impact of environmental variables and the preferences of the respondents. For example, the diversity and variability of habitats also affect the biodiversity of the river to a large extent, and these effects are connected with the river basin and its landscapes [31]. However, the global transition from undisturbed landscapes to human-dominated landscapes has affected ecosystems around the world and made the quantification of LULC an important indicator for evaluating the state of ecosystems [50]. Human activities on the landscape scale disrupt the process of maintaining the riverscape and its related biota, and often lead to habitat degradation and reduced heterogeneity [31]. These all directly or indirectly lead to the reduction of the social value for ES of the original natural landscape in the river basin. Therefore, some issues such as how to preserve the original riverscape as much as possible, how to maintain the original natural landscape of the river basin, and how to ensure that the social value for ES is not reduced are worthy of more in-depth research.

### 4.2. Thoughts on Landscape Construction

When people obtain social value, they always cause varying degrees of damage to the ecosystem [51]. Ecosystem services are the benefits that people obtain from the ecosystem [6], but due to the long-term use of unreasonable methods of obtaining these services by humans, the ecosystem is constantly being destroyed, which severely reduces its ability to provide services [31]. For example, in order to obtain greater economic benefits, people constantly change the types of land used, which threatens biodiversity and breaks the inherent balance of ecosystems [52]. Therefore, it is necessary to explore some approaches to landscape construction from an ecological perspective to make the ecosystem and its services sustainable, and to achieve harmony between man and nature.

Geographical factors affect the intensity and diversity of social values [45]. Taking the contribution of environmental variables to social value as a reference, these geographical factors can be used effectively. This will reduce the damage to the ecosystem caused by the drastic changes in geographic factors. Based on the evaluation results of the social value for ES on the east bank of the Fenghe River, we can come up with some valuable suggestions for landscape construction. For the aesthetic, recreation, and therapeutic values, the configuration of the landscape type and the distance between the landscape space and the river should be considered, and the influence of traffic factors should not be ignored. The space close to the river has a certain aesthetic value [49], and the existence of water also provides designers with more inspiration for the setting of healthcare facilities and entertainment spaces. This is conducive to improving the recreation and therapeutic value [48]. Through statistics on the contribution of environmental variables to social value, the reasonable configuration of landscape types is a key factor in promoting the aesthetic and therapeutic value. Based on near-water conditions, the aesthetic, recreation, and therapeutic values can be improved by the rational use of squares, forest landscapes, and wetland landscapes. In addition, convenient transportation not only helps to improve overall satisfaction [32], but also provides convenient conditions to experience various social values. For the historic value, the impact of DTW and DTR is small, while the contribution of landscape types is great. Therefore, when enhancing the historical value of a landscape, the original features (relics) should be protected.

In addition to referring to the contribution of environmental variables to enhance social value, we also need to choose some reasonable methods of landscape construction. Studies have shown that people like spaces with good natural environments [53], such as waterscape spaces with good water quality [54]; thus, we should retain their natural attributes. Usually, people’s tourism purpose is to appreciate the unique ecological landscape in the natural environment or the culture in the historical reserve [53], which may be the most valuable contribution that the landscape space must provide [55,56]. However, many landscape designs emphasize visual appeal and adopt uniform aesthetic rules [42], which makes the space lose their most unique value. In addition, humans can also take ecological measures to restore the natural environment of the landscape space [50], such as using more green infrastructure [57]. These are worthy of further study.

## 5. Conclusions

This paper takes the 4000-m buffer zone on the east bank of the Fenghe River as an example to evaluate the social values for the ES of the urban riverfront space under the influence of different environmental variables. The results show that the order of the M-VI of the seven social value types produced by the DTW model, DTR model, and DTW‒DTR‒LT model is aesthetic > recreation = therapeutic > historic > economic = future > spiritual, and the order produced by the LT model is aesthetic > recreation > therapeutic = historic > economic = future > spiritual. At the same time, we also found that different environmental variables have different effects on the distribution of social values. For example, when DTW is the only environmental variable, the social value presents an obvious striped distribution characteristic, but when DTR is the only variable, the social value is concentrated along the road. It is worth noting that when DTW, DTR, and LT are collectively used as environmental variables, the distribution pattern of social values is similar to when LT is used as the only variable; that is, various social values are mainly concentrated in the space where the 11 landscape types are located. In addition, the statistical results of MaxEnt show that LT makes a greater contribution to social value and is the factor that contributes the most to the aesthetic, therapeutic, and historic values, with contribution rates of 47.6, 50.5, and 80.0%, respectively. These results indicate that LT, as a special LULC, has a great influence on the social values of urban riverfront spaces. The other main findings of this research are: (1) DTW is the factor that contributes the most to the recreation value, with a contribution rate of 43.1%; (2) although the contribution of DTR to various social values is less than that of DTW or LT, its contribution to the aesthetic, recreation, and therapeutic values cannot be ignored; (3) hydrophilic landscapes including water, water‒plants, and water‒architectural are more popular with the respondents, and the average VI of various social values of these landscape spaces is higher.

In order to ensure that we can sustainably enjoy the benefits of an ecosystem, we should consider how to minimize the negative impact on the ecosystem when we construct landscapes to enhance social value. Therefore, this paper studies the contribution of environmental variables to social value, which can help us to conduct landscape construction under the premise of changing geographical factors as little as possible to improve the social value of ES in the space. This is to reduce unnecessary changes in geographic factors and further reduce the adverse impact on the natural ecosystem. In addition, some construction methods, such as how to rationally use green infrastructure or how to restore the destroyed ecological landscape, are worthy of more in-depth research.

## Figures and Tables

**Table 1:** Description of social value types for ecosystem services [20].

Social Value Type	Description
Aesthetic	I enjoy the scenery, sights, sounds, smells, etc.
Economic	It creates economic benefits for the region, and/or provides tourism opportunities.
Historic	It has places of natural and human historical significance that matter to me, others, or the nation.
Recreation	It provides a place for my favorite outdoor recreation activities.
Spiritual	It is a sacred, religious, or spiritually special place to me and/or I feel reverence and respect for nature there.
Therapeutic	It makes me feel better, physically and/or mentally.
Future	It allows future generations to know and experience it as it is now.

**Table 2:** Descriptions and sources of spatial data types.

Data Type		Description	Source
Study area boundary		Outer boundary of the study area	Using ArcGIS 10.2 to digitally process the 4000-m buffer zone on the east bank of the Fenghe River vector data.
Social value points		Social values assigned to places by respondents	Using ArcGIS 10.2 to digitally process the social value points obtained from the survey.
Environmental variables	Distance to Water	Distance to the east bank of Fenghe River	Obtained from the Fenghe vector data using the ArcGIS Euclidean distance tool
	Distance to Road	Distance to the main road in the study area	Obtained from the road vector data using the ArcGIS Euclidean distance tool
	Landscape Type	11 types of landscapes in the study area	Using ArcGIS 10.2 to visually interpret satellite images of the study area and convert them into raster data.

**Table 3:** The area under the receiver operating characteristic curve based on different environmental variables.

Social Value Type	Test AUC1	Test AUC2	Test AUC3	Test AUC4
Aesthetic	0.981	0.985	0.973	0.993
Economic	0.983	0.961	0.949	0.985
Historic	0.976	0.986	0.974	0.998
Recreation	0.986	0.975	0.973	0.989
Spiritual	0.979	0.986	0.970	0.995
Therapeutic	0.981	0.989	0.974	0.993
Future	0.981	0.950	0.920	0.985

Note: Test AUC1 is jointly generated from distance to water, distance to road, and landscape types; Test AUC2 is generated from distance to water; Test AUC3 is generated from distance to road; Test AUC4 is generated from landscape types.

**Table 4:** The average nearest statistics and the maximum value index (M-VI) of seven social value types.

Social Value Type	DTW		DTR		LT		DTW-DTR-LT	
	M-VI	R Value	M-VI	R Value	M-VI	R Value	M-VI	R Value
Aesthetic	10	0.0017	10	0.0017	9	0.0017	10	0.0017
Economic	5	0.0017	5	0.0017	5	0.0017	5	0.0017
Historic	7	0.0501	7	0.0501	7	0.0501	7	0.0501
Recreation	8	0.0018	8	0.0018	8	0.0018	8	0.0018
Spiritual	4	0.0026	4	0.0026	3	0.0026	3	0.0026
Therapeutic	8	0.0144	8	0.0144	7	0.0144	8	0.0144
Future	5	0.0019	5	0.0019	5	0.0019	5	0.0019

Note: DTW refers to distance to water as the only environmental variable; DTR refers to distance to road as the only environmental variable; LT refers to landscape type as the only environmental variable; DTW‒DTR‒LT means that distance to water, distance to road, and landscape type are collectively used as environmental variables; M-VI is the maximum value index; R value is the result of average nearest neighbor statistics for each social value type.

**Table 5:** Statistics of the value index of each landscape space.

Landscape Type	Aesthetic			Recreation			Therapeutic			Historic		
	Mean	Min	Max	Mean	Min	Max	Mean	Min	Max	Mean	Min	Max
Water	6.80	0	8	3.40	0	4	4.32	0	7	0.85	0	1
Water‒Plants	6.89	0	8	3.89	0	4	4.92	0	7	0.97	0	1
Water‒Architectural	6.32	6	8	4.00	4	4	5.00	5	5	1.00	1	1
Amusement Park	2.01	0	8	1.90	0	8	2.26	0	7	0.06	0	6
Square	4.76	0	8	3.36	0	8	2.00	0	5	1.09	0	3
Forest	2.9	0	7	0.51	0	4	2.41	0	5	0.01	0	1
Wetland	3.23	1	7	1.23	1	4	2.70	1	7	0.04	0	5
Beach	0.29	0	6	0.00	0	0	0.23	0	6	0.00	0	0
Relic	4.92	0	9	1.96	0	4	2.88	0	5	4.54	0	7
Commercial Street	4.81	0	7	3.43	0	5	1.28	0	4	1.77	0	6
Other Landscape	0.16	0	9	0.09	0	8	0.11	0	7	0.02	0	7

Note: Mean refers to the average value of the value index in a certain landscape space; Min refers to the minimum value of the value index in a certain landscape space; Max refers to the maximum value of the value index in a certain space.

**Table 6:** Spatial autocorrelation analysis between distance variables and value index.

Environmental Variable	Reference Item	Value Index of Aesthetic	Value Index of Recreation	Value Index of Therapeutic	Value Index of Historic
Distance to Water	Moran’s I	−0.061	−0.028	−0.068	0
	*p* value	0.001	0.001	0.001	0.467
	*Z* score	−31.4173	−14.5657	−35.1869	−0.1226
Distance to Road	Moran’s I	0.068	0.084	0.071	0.091
	*p* value	0.001	0.001	0.001	0.001
	*Z* score	35.3432	43.8966	36.7976	46.6648

Note: *p* value represents the probability; Z score represents the multiple of the standard deviation. When |Z| > 1.65 and *p* < 0.10, the confidence level is 90%; when |Z| > 1.96 and *p* < 0.05, the confidence level is 95%; when |Z| > 2.58, *p* < 0.01, the confidence level is 99%.

**Table 7:** The contribution of environmental variables to social value.

Social Value Type	Contribution of Distance to Water	Contribution of Distance to Road	Contribution of Landscape Type
Aesthetic	30.5%	21.9%	47.6%
Recreation	43.1%	17.8%	39.1%
Therapeutic	29.3%	20.2%	50.5%
Historic	9.9%	10.1%	80.0%

**Figure A1:**
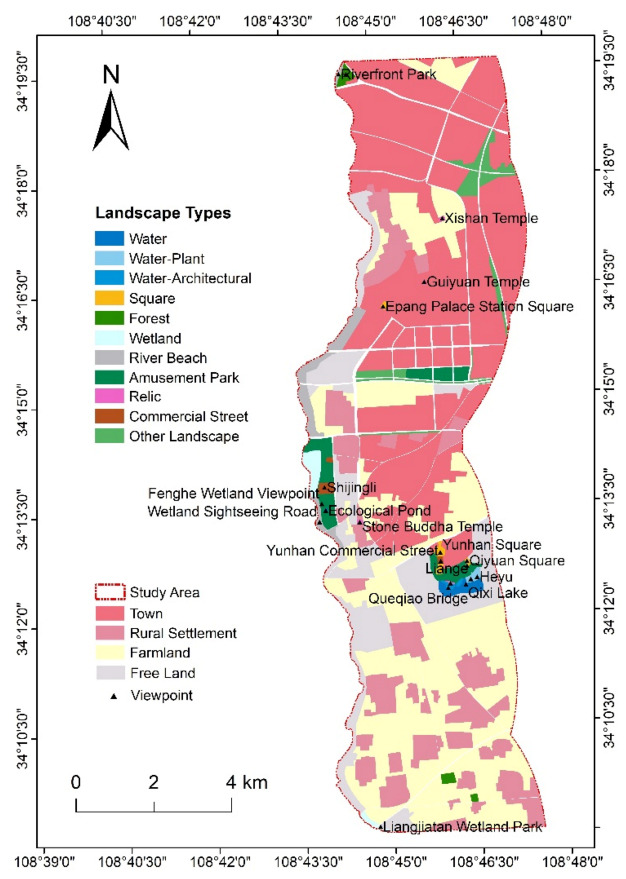
Location of scenic spots on the east bank of Fenghe River.

## Data Availability

The data presented in this study are available on request from the corresponding author. The data are not publicly available due to part of them are being used in other studies that have not yet been publicly published.

## Appendix A

**Questionnaire about the Evaluation of Social Values for Ecosystem Services in Urban Riverfront Space Based on the SolVES Model: A Case Study of the Fenghe River, Xi’an, China**

Part 1. Traveling characteristics and satisfaction survey

Q1: In the past five years, how often have you visited (walking, traveling, participating in group activities, etc.) a scenic site or other landscape on the east bank of the Fenghe River? (Single choice)

**A.** _____times/week **B.** _____times/month

**C.** _____times/year **D.** first time/just once

Q2: Generally, what were your reasons (purposes) for traveling on the east bank of the Fenghe River? (Multiple choice)

**A.** To enjoy the landscape  ** B.** To participate in entertainment activities

**C.** For religious reasons **D.** For sports

**E.** For a picnic or party **F.** Other purpose_________________

Q3: Who do you prefer to travel with in general? (Multiple choice)

**A.** Travel alone **B.** Family **C.** Classmates **D**. Colleagues **E.** Other

Q4: In which season(s) do you like to travel to the east bank of the Fenghe River (Multiple choice):

**A.** Spring **B.** Summer **C.** Autumn **D.** Winter

Q5: During your travels, are you satisfied with the tourism-related infrastructure on the east bank of the Fenghe River? (Including guidance, transportation, shopping facilities, etc.) (Single choice)

**A.** Very satisfied **B.** Satisfied **C.** Neutral **D.** Dissatisfied **E.** Very dissatisfied

Q6: During your travels, are you satisfied with the traveling environment on the east bank of Fenghe River? (Including safety, crowdedness of scenic spots, price of scenic spots, etc.) (Single choice)

**A.** Very satisfied **B.** Satisfied **C.** Neutral **D.** Dissatisfied **E.** Very dissatisfied

Q7: What do you think of the attractiveness of the east bank of the Fenghe River? (Including nature, culture, entertainment, etc.) (Single choice)

**A.** Very satisfied **B.** Satisfied **C.** Neutral **D.** Dissatisfied **E.** Very dissatisfied

Q8: Are you satisfied with the overall travel experience of the east bank of the Fenghe River? (Single choice)

**A.** Very satisfied **B.** Satisfied **C.** Neutral **D.** Dissatisfied **E.** Very dissatisfied

Q9: In your opinion, which aspects of this area are particularly in need of improvement? (Optional)

Part 2. Value allocation and marking of social value points.

Q10: We would like to know your feelings about the social value of the east bank of the Fenghe River. Assume that you can spend 100 yuan to guarantee the existing value of the east bank of the Fenghe River. You can allocate the money to the following seven social values in accordance with your personal preferences, but your total cost cannot be over 100 yuan. You may give 100 yuan to just one social value, but then you will have no extra money to allocate to other social values; or you may, e.g., allocate 50 yuan to one value, 25 yuan to another, and 25 yuan to other values. Please remember, the total amount you spend must be 100 yuan.

**￥____ Aesthetic:** I enjoy the scenery, sights, sounds, smells, etc.

**￥____ Economic:** It creates economic benefits for the region, and/or provides tourism opportunities.

**￥____ Historic:** It has places and things of natural and human historical value that matter to me, others, or the nation.

**￥____ Recreation:** It provides a place for my favorite outdoor recreation activities.

**￥____ Spiritual:** It is a sacred, religious, or spiritually special place to me and/or I feel reverence and respect for nature there.

**￥____ Therapeutic:** It makes me feel better, physically and/or mentally.

**￥____ Future:** It allows future generations to know and experience it as it is now.

Q11: Which scenic spots or spaces on the east bank of the Fenghe River do you think can represent the social value you selected just now? Please mark them in the table (Table A2) or on the map (Figure A1). For your convenience, we have listed 20 scenic spots on the east bank of the Fenghe River (Table A1), but your selection is not limited to these. You can mark the places that you think are important on the map, and note their names and corresponding value types.

**Table A1:** The main scenic spots on the east bank of Fenghe River and their numbers.

Number	Place Name	Number	Place Name	Number	Place Name	Number	Place Name
1	Riverfront Park	6	Shijingli	11	Qixi Lake	16	Yunhan Commercial Street
2	Fenghe Forest Park	7	Fenghe Wetland Viewpoint	12	Queqiao Bridge	17	Kunmingchi Remains
3	Xishan Temple	8	Stone Buddha Temple	13	Liangjiatan Wetland Park	18	Qiyuan Square
4	Guiyuan Temple	9	Yunhan Square	14	Wetland Sightseeing Road	19	Heyu
5	Epang Palace Station Square	10	Children Playground	15	Ecological Pond	20	Liange

**Table A2:** Social value point collection.

Social Value types	Place Name or Number
Aesthetic	
Economic	
Historic	
Recreation	
Spiritual	
Therapeutic	
Future	

Part 3. Basic information of Respondents

Q12: Your gender is:

**A.** Male **B.** Female

Q13: Your age is:

**A.** 0–6 years old **B.** 7–17 years old **C.** 18–40 years old

**D.** 41–65 years old **E.** Over 65 years old

Q14: Your highest educational background is:

**A.** Lower than high school **B.** High school

**C.** Technical/Vocational **D.** Bachelor’s degree

**E.** Postgraduate or above

Q15: Your monthly income is:

**A.** Less than 3000 yuan **B.** 3000–5000 yuan **C.** 5000–10,000 yuan

**D.** 10,000–20,000 yuan **E.** Over 20,000 yuan

Q16: Your occupation is:

**A.** General staff **B.** Commercial or service personnel

**C.** Enterprise management personnel **D.** Individual practitioner

**E.** Civil servant **F.** Student

**G.** Science, education, culture, or health practitioner

**H.** Soldier **I.** Other occupation_____  

Q17: You come from:

**A.** Xi’an/Xianyang **B.** Other regions in Shaanxi Province

**C.** Other regions of China **D.** Other countries

## Appendix B

**Multicollinearity analysis of environmental variables**

If there is multicollinearity between environmental variables, the results of AUC may be unreliable [35], so we conducted multicollinearity tests on DTW, DTR and LT. In order to ensure a more reliable test result, we adopted three methods to test multicollinearity, including the Pearson correlation coefficient, variance inflation factors, and eigenvalue and condition index. The specific results are as follows.

(1) Pearson correlation coefficient

It can be seen from Table A3 that DTR and DTW are significantly negatively correlated with VI, and LT and VI are significantly positively correlated. Generally, when the Pearson correlation coefficient (PCC) is greater than 0.7, there may be collinearity between the corresponding two variables [58]. The result shows that the PCC between DTW and DTR is 0.826, which indicates that they may be collinear. However, because the PCC only considers the correlation between the two variables, and the correlation between the variables is intricate, it is necessary to conduct further tests [59].

**Table A3:** Correlation coefficient matrix of distance to water, distance to road, landscape types and value index.

Variable	Reference Item	Distance to Road	Distance to Water	Landscape Type	Value Index
Distance to Road	Pearson Correlation	1	0.826 **	−0.321 **	−0.132 *
	Sig. (2-tailed)		0.000	0.000	0.018
	N	324.000	324.000	324.000	324.000
Distance to Water	Pearson Correlation	0.826 **	1	−0.134 *	−0.300 **
	Sig. (2-tailed)	0.000		0.015	0.000
	N	324.000	324.000	324.000	324.000
Landscape Type	Pearson Correlation	−0.321 **	−0.134 *	1	0.115 *
	Sig. (2-tailed)	0.000	0.015		0.039
	N	324.000	324.000	324.000	324.000
Value Index	Pearson Correlation	−0.132 *	−0.300 **	0.115 *	1
	Sig. (2-tailed)	0.018	0.000	0.039	
	N	324.000	324.000	324.000	324.000

Note: ** Correlation is significant at the 0.01 level (2-tailed). * Correlation is significant at the 0.05 level (2-tailed).

(2) Variance inflation factors

When the tolerance is greater than 0.1 and the variance inflation factors (VIF) are less than 5, it indicates that there is no collinearity between the variables [60]. As shown in Table A4, the variance inflation factors (VIF) of DTW, DTR, and LT are all less than 5, and the tolerances are all greater than 0.1, which indicates that these variables meet the modeling standards.

**Table A4:** Collinearity statistics of distance to water, distance to road, and landscape types.

Environmental Variable	Collinearity Statistics	
	Tolerance	Variance Inflation Factor
Distance to Road	0.273	3.663
Distance to Water	0.299	3.345
Landscape Type	0.843	1.186

Note: The greater the value of the variance inflation factor (VIF), the more significant the multicollinearity; when VIF < 10, it is considered that there is no multicollinearity; when VIF > 10, the multicollinearity is significant; when the tolerance index is less than 0.1, the model multicollinearity is significant.

(3) Eigenvalue and condition index

According to the collinearity diagnostic criteria of Zhang W.T. (2013), when the eigenvalue of a dimension is equal to 0, or the condition index of a dimension is greater than 30, there may be collinearity [60]. As shown in Table A5, the eigenvalues of the four dimensions are not equal to 0, and the values of the condition index are all less than 30, which indicates that there is no multicollinearity between the variables.

**Table A5:** Multidimensional statistics of eigenvalue and condition index.

Dimension	Eigenvalue	Condition Index	Variance Proportions		Distance to Water	Landscape Type
			(Constant)	Distance to Road		
1	3.522	1.000	0.010	0.000	0.010	0.010
2	0.366	3.103	0.010	0.030	0.060	0.220
3	0.082	6.545	0.370	0.060	0.320	0.380
4	0.030	10.892	0.610	0.910	0.610	0.390

Note: Dependent variable is value index (VI); when the value of eigenvalue of a dimension is equal to 0, there may be serious collinearity; when the condition index of a dimension is greater than 30, there may be collinearity.

Based on the results of VIF, eigenvalue, and condition index, we could judge that there is no multicollinearity among the environmental variables (distance to water, distance to road, and landscape type) selected in this paper.

## References

[1] Burkhard B., Kroll F., Nedkov S., Müller F. (2012). Mapping ecosystem service supply, demand and budgets. Ecol. Indic..

[2] (1970). SCEP Study of critical environmental problems. Man’s Impact on the Global Environment: Assessment and Recommendations for Action.

[3] Grasdalen H., Eriksson L.E.G., Westman J., Ehrenberg A. (1977). Surface potential effects on metal ion binding to phosphatidylcholine membranes. 31 P NMR Study Lanthan. Calcium Ion Bind. Egg-Yolk Lecithin Vesicles.

[4] Ehrilich P.R., Ehrilich A.H. (1981). Extinction: The Causes and Consequences of the Disappearance of Species.

[5] Costanza R., Arge R., De Groot R., Farber S., Grasso M., Hannon B., Limburg K., Naeem S., O’Neill R.V., Paruelo J. (1997). The value of the world ecosystem services and natural capital. Nature.

[6] Daily G.C. (1997). Nature’s Services: Societal Dependence on Natural Ecosystems.

[7] Carpenter S.R., Pingali P.L., Bennett E.M. (2005). Ecosystems and Human Well-Being.

[8] Haines-Young R.H. (2011). Exploring ecosystem service issues across diverse knowledge domains using Bayesian Belief Networks. Prog. Phys. Geogr..

[9] Wallace K.J. (2007). Classification of ecosystem services: Problems and solutions. Biol. Conserv..

[10] Bennett E.M., Peterson G.D., Gordon L.J. (2009). Understanding relationships among multiple ecosystem services. Ecol. Lett..

[11] MEA Millennium Ecosystem Assessment (2003). Ecosystems and Human Well-Being: A Framework for Assessment.

[12] Carpenter S.R., Mooney H.A., Agard J., Capistrano D., Defries R.S., Diaz S., Dietz T., Duraiappah A.K., Oteng-Yeboah A., Pereira H.M. (2009). Science for managing ESs: Beyond the millennium ecosystem assessment. PNAS.

[13] Teller A. (2016). Presentation for the European MAES Working Group. MAES-Related Activities in MS 2014–2015, 8 December 2014.

[14] Maes J., Teller A., Erhard M., Liquete C., Braat L., Berry P., Egoh B., Puydarrieux P., Fiorina C., Santos F. (2013). An Analytical Framework for Ecosystem Assessments under Action 5 of the EU Biodiversity Strategy to 2020.

[15] Schröter M., Albert C., Marques A., Tobon W., Lavorel S., Maes J., Brown C., Klotz S., Bonn A. (2016). National Ecosystem Assessments in Europe: A Review. BioScience.

[16] Liquete C., Kleeschulte S., Dige G., Maes J., Grizzetti B., Olah B., Zulian G. (2015). Mapping green infrastructure based on ecosystem services and ecological networks: A Pan-European case study. Environ. Sci. Policy.

[17] Sherrouse B.C., Semmens D.J. (2014). Validating a method for transferring social values of ESs between public lands in the Rocky Mountain region. Ecosyst. Serv..

[18] Brown G., Brabyn L. (2012). The extrapolation of social landscape values to a national level in New Zealand using landscape character classification. Appl. Geogr..

[19] Sherrouse B.C., Semmens D.J. Social Values for Ecosystem Services, Version 2.0 (SolVES 2.0): Documentation and User Manual. https://pubs.usgs.gov/of/2012/1023/contents/OF12-1023.pdf.

[20] Sherrouse B.C., Clement J.M., Semmens D.J. (2011). A GIS application for assessing, mapping, and quantifying the social values of ecosystem services. Appl. Geogr..

[21] Riper C.J.V., Kyle G.T., Sutton S.G., Barnes M., Sherrouse B.C. (2012). Mapping outdoor recreationists’ perceived social values for ecosystem services at Hinchinbrook Island National Park, Australia. Appl. Geogr..

[22] Brown G., Pullar D., Hausner V. (2016). An empirical evaluation of spatial value transfer methods for identifying cultural ESs. Ecol. Indic..

[23] Brander L.M., Wagtendonk A.J., Hussain S.S., Mcvittie A., Verburg P.H. (2012). Ecosystem service values for mangroves in Southeast Asia: A meta-analysis and value transfer application. Ecosyst. Serv..

[24] Chaikumbung M., Doucouliagos H., Scarborough H. (2016). The economic value of wetlands in developing countries: A meta-regression analysis. Ecol. Econ..

[25] Ghermandi A., Sheela A.M., Justus J. (2016). Integrating similarity analysis and ecosystem service value transfer: Results from a tropical coastal wetland in India. Ecosyst. Serv..

[26] Zhang H., Gao Y., Hua Y., Zhang Y., Liu K. (2019). Assessing and mapping recreationists’ perceived social values for ecosystem services in the Qinling Mountains, China. Ecosyst. Serv..

[27] Wang Y., Fu B., Lyu Y.P., Yang K., Che Y. (2016). Assessment of the social values of ecosystem services based on SolVES model: A case study of Wusong Paotaiwan Wetland Forest Park. Chin. J. Appl. Ecol..

[28] Zhao Q., Li J., Liu J., Qin K., Tian T. (2018). Assessment and analysis of social values of cultural ecosystem services based on the solves model in the Guanzhong-Tianshui Economic Region. Acta Ecol. Sin..

[29] Cheng D.Y., Li M.T., Ding Y.Y., Che Y. (2018). Assessment of the urban waterfront based on social values of ecosystem services: A case study of the Huangpu River waterfront. Shanghai Urban Plan. Rev..

[30] Gao Y., Liu K., Ma Q., Li Y., Fan Y.-N., Li X.-Q., Gu C. (2017). Assessment of the social value of ecosystem services based on SolVES model and visitor’s preference: A case study of Taibai Mountain National Forest Park. Chin. J. Ecol..

[31] Allan J.D. (2004). LANDSCAPES AND RIVERSCAPES: The Influence of Land Use on Stream Ecosystems. Annu. Rev. Ecol. Evol. Syst..

[32] Karen T., Peter S. (2007). An Investigation of the relationship between public transport performance and destination satisfaction. J. Transp. Geogr..

[33] Zhang F.P., Zhao S., Zhou Z.C., Wei Y.F. (2014). Relationship between changes of land use pattern and water quality in Fenghe River Basin. Bull. Soil Water Conserv..

[34] Ma Q., Liu K., Gao Y., Li Y., Fan Y.N., Gu C. (2018). Assessment on social values of ecosystem services in Xi’an Chanba National Wetland Park based on SolVES model. Wetl. Sci..

[35] Sherrouse B.C., Semmens D.J. Social Values for Ecosystem Services, version 3.0 (SolVES 3.0): Documentation and User Manual. https://pubs.usgs.gov/of/2015/1008/pdf/ofr2015-1008.pdf.

[36] Wetherbee G.A., Debey T.M., Nilles M.A., Lehmann C.M.B., Gay D.A. (2012). Social Values for Ecosystem Services, Version 2.0 (SolVES 2.0): Documentation and User Manual.

[37] Phillips S.J., Anderson R.P., Schapire R.E. (2013). Maximum entropy modeling of species geographic distributions. Ecol. Model..

[38] Hosmer D.W., Lemeshow S. (2000). Applied Logistical Regression.

[39] Swets J.A. (1988). Measuring the accuracy of diagnostic systems. Science.

[40] Huang C.H., Yang J., Zhang W.J. (2013). Development of ecosystem services evaluation models: Research progress. Chin. J. Ecol..

[41] Sherrouse B.C., Semmens D.J., Clement J.M. (2014). An application of Social Values for Ecosystem Services (SolVES) to three national forests in Colorado and Wyoming. Ecol. Indic..

[42] Sun F.Y., Xiang J.Y., Tao Y., Tong C., Che Y. (2019). Mapping the social values for ecosystem services in urban green spaces: Integrating a visitor-employed photography method into SolVES. Urban For. Urban Green..

[43] Plieninger T., Dijks S., Oteros-Rozas E., Bieling C. (2013). Assessing, mapping, and quantifying cultural ecosystem services at community level. Land Use Policy.

[44] Larson L.R., Keith S.J., Fernandez M., Hallo J.C., Shafer C.S., Jennings V. (2016). Ecosystem services and urban greenways: What’s the public’s perspective?. Ecosyst. Serv..

[45] Ives C.D., Oke C., Hehir A., Gordon A., Wang Y., Bekessy S.A. (2017). Capturing residents’ values for urban green space: Mapping, analysis and guidance for practice. Landsc. Urban Plan.

[46] Swanwick C. (2009). Society’s attitudes to and preferences for land and landscape. Land Use Policy.

[47] Kaplan R., Kaplan S. (1989). The Experience of Nature: A Psychological Perspective.

[48] Preussner D.C. (2010). The Sustainable Landscaping: Recycling Materials Water Conservation.

[49] Calkins M. (2008). Materials for Sustainable Sites: A Complete Guide to the Evaluation, Selection, and Use of Sustainable Construction Materials.

[50] Grübler A., Meyer W.B., Turner B.L. (1994). Changes in Land Use and Land Cover: A Global Perspective.

[51] Maes J., Jacobs S. (2017). Nature-Based Solutions for Europe’s Sustainable Development. Soc. Conserv. Biol..

[52] Quinn J.M., Brown P.M., Boyce W., Mackay S., Taylor A., Fenton T. (2001). Riparian zone classification for management of stream water quality and ecosystem health. J. Am. Water Resour. Assoc..

[53] Zhang X., Zhang Q. (2021). Improvement of the construction of sociecological environment and enhancement of subjective well-being of urban residents. J. Liaoning Norm. Univ..

[54] Herzog T.R. (1985). A cognitive analysis of preference for waterscapes. J. Environ. Psychol.

[55] Chiesura A. (2004). The role of urban parks for the sustainable city. Landsc. Urban Plan.

[56] Gobster P.H., Nassauer J.I., Daniel T.C., Fry G. (2007). The shared landscape: What does aesthetics have to do with ecology?. Landsc. Ecol..

[57] Maes J., Barbosa A., Baranzelli C., Zulian G., de Silva F.B., Vandecasteele I., Hiederer R., Liquete C., Paracchini M.L., Mubareka S. (2015). More green infrastructure is required to maintain ecosystem services under current trends in land-use change in Europe. Landsc. Ecol..

[58] Lu M. (2007). A comparative study of several methods for dealing with multicollinearity. TJYJC.

[59] Zhu Y., Zheng Y., Yin M. (2020). Multicollinearity Test Under Statistical Significance. TJYJC.

[60] Zhang W.T. (2013). Advanced Tutorial on SPSS Statistical Analysis.

